# Prevalence and antimicrobial resistance profiles of *Campylobacter coli*, *Campylobacter jejuni*, and *Escherichia coli* in the poultry distribution network

**DOI:** 10.3389/frabi.2026.1875259

**Published:** 2026-09-22

**Authors:** Sadik Dantroliya, Monica Chavan, Debashrita Mittra, Rameshchandra Pandit, Fiona Tomley, Damer Blake, Prakash Koringa, Chaitanya Joshi, Satyamitra Shekh, Chinmayi Joshi, Madhvi Joshi

**Affiliations:** 1 Gujarat Biotechnology Research Centre, Department of Science and Technology, Government of Gujarat, Gandhinagar, Gujarat, India; 2 Smt. S. S. Patel Nootan Science and Commerce College, Sankalchand Patel University, Visnagar, Gujarat, India; 3 The Royal Veterinary College, Hatfield, United Kingdom; 4 Department of Veterinary Biotechnology, College of Veterinary Science and Animal Husbandry, Kamdhenu University, Anand, Gujarat, India

**Keywords:** *Campylobacter coli*, *Campylobacter jejuni*, *Escherichia coli*, poultry farms, broiler, desi chicken

## Abstract

India is one of the largest producers of poultry and poultry-derived products. The dynamics of animal production and consumer exposure have been linked to new challenges in the control of poultry-borne zoonotic diseases as consumption of poultry products raises. As a part of the One Health Poultry Hub, this study focused on addressing the risk posed by pathogens associated to the intensification of poultry production. Eight cities in the state of Gujarat were included in the study based on poultry density and distribution networks (PDN). Samples were collected from 65 live bird market shops and 50 poultry farms. The prevalence of key food borne pathogens - specifically *Campylobacter* spp. and *Escherichia coli -* was assessed using cecal and cloacal samples from birds, while non-typhoidal *Salmonella* (NTS) spp. was examined in environment samples from poultry farms. *Campylobacter* spp. and *E. coli* were recovered from 10.26% and 51.33% birds, respectively, whereas NTS spp. was not detected in any environmental sample. Prevalence pattern varied by city, season and breed. The highest *C. coli* prevalence was in broiler shop samples from Surat (17.5%) and desi chickens form Bhuj (16.7%), while *C. jejuni* peaked in broiler shop samples from Bhuj (13.3%). *C. jejuni* was not detected in desi chickens. All isolates were further analyzed for antimicrobial susceptibility and resistance using the disc diffusion method. A higher resistance was observed in *Campylobacter* isolates (55.74%) particularly against fluoroquinolones, macrolides and beta-lactams. Among the isolated organisms, 39.34% of *Campylobacter* and 61.03% of *E. coli* isolates exhibited extensively drug-resistant (XDR) phenotypes, while 4.92% of *Campylobacter* and 9.09% of *E. coli* demonstrated pan drug-resistant (PDR) phenotypes. Among *Escherichia coli* isolates, 29.87% exhibited resistance to glycopeptides, nitrofurantoin, macrolides, tetracycline, beta-lactams, and fluoroquinolones. These findings underscore the critical need for implementing farm hygiene practices to mitigate the risk of food borne pathogens and antimicrobial resistance within poultry distribution networks.

## Introduction

The G20 initiative promoting the ‘One Earth, One Health’ approach has elevated the One Health concept to a global research priority, emphasizing its role in combating pandemics, ecosystem balance, and achievement of the Sustainable Development Goals. In this context, mapping food safety risks and antimicrobial resistance (AMR) emerging from the human-animal-environment interfaces is essential for public health management worldwide. Globally, foodborne pathogens infect approximately 7.7% of the population annually-contributing to 7.5% of global deaths with 31 types of foodborne illnesses causing up to 600 million disease outbreaks each year (Albert et al., 2024; Havelaar et al., 2015). As a major livestock-rearing nation, India faces a high risk of zoonotic infections acquired from animals, environment, carriers or infected patients, and contaminated food. Notably, 60% of emerging and re-emerging pathogens in India are transmitted from food and environment sources to humans (Albert et al., 2024). The Integrated disease surveillance program by the National Centre for Disease Control (NCDC) reported 15,455 disease outbreaks across India between 2008 to 2017 (https://ncdc.mohfw.gov.in/). Among these, 2688 foodborne disease outbreaks were studied to identify vehicles, of which 58 were associated with meat, poultry and eggs (Bisht et al., 2021). Gujarat, being one of the states ranking top in the highest reported average outbreaks (>10) as per the study by Bisht et al. (2021), requires systematic surveillance and monitoring of pathogenic and antimicrobial-resistant organisms in the human-animal-environment chain to support outbreak investigation and analysis.

Several factors such as high farm density, poor waste management, and inadequate animal handling practices contribute to increased risk of infection spread (Xin et al., 2025). Zoonotic spread has been reported earlier for disease-causing AMR pathogens from various farm animals to humans (Dafale et al., 2020; Olaru et al., 2023). Major food-borne pathogens such as *Campylobacter* spp., nontyphoidal *Salmonella* (NTS) and *E. coli* inhabit the gastrointestinal tract of poultry and other animals. *Campylobacter* spp. - a leading cause of diarrheal disease worldwide affecting both children and adults - is commonly found in the gastrointestinal tracts of domestic animals (cattle, goats, sheep, pigs, and poultry) as well as in wild birds and other animals (Endtz, 2020; Schweitzer et al., 2021; Libera et al., 2022; Paintsil et al., 2022; Sahoo et al., 2022). Campylobacteriosis in humans occurs mainly through the ingestion of contaminated food, along with handling and consuming raw or undercooked poultry meat (Wysok et al., 2024). The bacteria primarily inhabit the cecum and colon of avian species, with cell counts ranging from 10^6^ to 10^8^ (Lu et al., 2021). The high prevalence of *Campylobacter*, NTS and *E. coli* species in broiler chicken meat renders it a major source of these pathogens to the consumers (Thames and Sukumaran, 2020). It has been reported that in the natural environment up to 15% of coliform bacteria residing in the intestines have the potential to act as opportunistic pathogens (Sampson et al., 2020). Infection with pathogenic strains of *E. coli* adversely affects immunocompromised hosts leading to septicemia, endocarditis, diarrhea, meningitis and urinary tract infections (Kumar et al., 2020). Inappropriate antibiotic use is a primary driver of emergence and dissemination of AMR strains in both humans and animals (Bista et al., 2020). Nontyphoidal *Salmonella* cause an estimated 153 million annual cases of salmonellosis and 57,000 deaths worldwide, with AMR being a major concern for the treatment (Elnour et al., 2022; Jajere, 2019; Sharma et al., 2019; Walia et al., 2019). Recently in Gujarat, *S.* Typhi outbreak was reported and genome analysis revealed notable levels and mechanisms of AMR spread (Akshay et al., 2025). Given the preference of Indian consumers for fresh, slaughtered poultry birds, the probability of cross-contamination of poultry carcasses increases. Despite the high outbreak burden, systematic baseline surveillance data on the prevalence and AMR profiles of major foodborne pathogens across the poultry value chain in Gujarat remain scarce. Previous studies in the region have been largely outbreak-driven, limited to single pathogens, or restricted to clinical isolates from hospitalized patients, with little attention to community-level farm and market nodes. Dantroliya et al. (2025) previously reported AST and genomic data for 18 *Campylobacter* isolates; these 18 are included as a subset of the 77 isolates characterized in the present comprehensive surveillance across eight Gujarat cities. Moreover, concurrent evaluation of *Capmylobacter* species, non-typhoidal *Salmonella* and *E*. *coli* within the same sampling framework has not been performed in this geographic context. To address this gap, the present study focuses to (i) estimate the prevalence of these three pathogens across poultry farms and live bird shops in eight major cities of Gujarat, (ii) characterize their phenotypic AMR profiles against clinically and veterinary relevant antibiotics, and (iii) generate a baseline evidence to inform region-specific AMR surveillance and intervention strategies under the One Health umbrella.

## Materials and methods

### Study design and sampling framework

As a part of GCRF-One Health Poultry Hub project, poultry distribution networks (PDNs) for broilers and desi (indigenous) reared chickens in Gujarat were mapped (Pelligand et al., 2024). A total of 50 farms and 65 live bird shops; belonging to these PDNs across eight cities from Gujarat were included for sample collection based on the willingness of the farm and shop owners.

### Sample collection

From each study site, five broiler and/or desi chickens were selected based on healthy appearance and body weight (broiler: > 1 kg; desi breed: 0.8–1 kg). Birds exhibiting clinical signs of illness or with body weights below the established breed-specific reference standards were excluded from the study. All birds were euthanized by manual cervical dislocation and dissected for the collection of cloacal and cecal samples. A total of 750 chicken ceca samples were collected, both the ends of each cecum were tied with sterile thread, and stored in sterile phosphate buffered saline (PBS, pH 7.0) at 4°C until further processing for bacterial isolation. For *Escherichia coli* isolation, 150 swabs per study site were collected from bird-1 and transported in Brain Heart Infusion (BHI, HiMedia, India) broth at 4°C. For the isolation of non-typhoidal *Salmonella* spp., environmental swabs (litter samples from chicken cage surface in shops and soil surfaces in poultry farms) were collected into BHI and transported to the laboratory at 4°C. A pre-designed coding system was used for site, bird and sample type. Upon receipt, all samples were stored at 0-4°C until further processing for the culture isolation. From August 2021 to March 2022, a total of 750 samples for *Campylobacter* species, 150 for *E. coli* and 150 for non-typhoidal *Salmonella* were collected. Each isolate was assigned a unique ID number e.g., 100-01–1 where first three digits denote the site ID (farm or live bird shop), the next two digits denote the bird number and final digit denotes the isolate ID (**Supplementary Table 1**). The required sample size for *Campylobacter* spp. surveillance was initially estimated based on expected prevalence of 10%, 5% absolute precision, and a 95% confidence level. Considering the multi-city surveillance design and the need to capture geographical variation, a total of 750 cecal samples were collected from eight cities in Gujarat. For *E. coli* and NTS, 150 samples each were collected based on feasibility and resource constrains, consistent with similar poultry surveillance studies.

### Isolation of *Campylobacter* spp.

*Campylobacter* isolates were recovered from the chicken cecal samples as per International Standards Organization (ISO) protocols ISO 10272-1:2017 (International Organization for Standardization, 2017). Inside a biosafety cabinet, each cecum was opened. The cecal content was removed and then inner mucosal surface test portion was obtained using a sterile cotton swab. In strict accordance with ISO 10272-1:2017, the swab was immediately streaked onto modified charcoal cefoperazone deoxycholate agar (mCCDA, Oxoid, UK), a blood-free selective medium (Ugarte-Ruiz et al., 2012). Plates were incubated under microaerophilic conditions (5% CO_2_) at 42 °C for 48 hours to selectively inhibit competing microbes while promoting *Campylobacter* growth, consistent with ISO 10272-1:2017 recommendations for poultry samples (Dantroliya et al., 2025). Visible grayish colonies observed on plates were purified by sub culturing on mCCDA plates.

### Isolation of non-typhoidal *Salmonella* spp.

For isolation of *Salmonella* spp., environmental swab samples collected as per Abey et al. (2024), were inoculated into BHI broth and incubated at 37°C for 48 hours. After visible growth, appropriate dilutions (10^-4^-10^-6^) were prepared and spread on Modified Semisolid RV (MSRV) agar plates (Himedia, India) and incubated (Tietjen and Fung, 1995). Colonies with a zone of clearance were streaked onto Xylose Lysine Deoxycholate (XLD) agar plates (Himedia, India). Colonies with black centers were further studied for culture identification.

### Isolation of *Escherichia coli*

For *E. coli* isolation, cloacal swabs were inoculated into BHI broth and incubated at 37°C for 24 hours. Appropriate dilutions were then spread on MacConkey’s agar plates (Himedia, India). After overnight incubation, pink colored colonies were streaked onto Eosin Methylene Blue agar plates (Himedia, India) (Leininger et al., 2001). Colonies with greenish metallic sheen were studied further.

### Biochemical characterization of isolates

Species specific biochemical tests were performed including the indole test, methyl red test, Voges-Proskauer test, and citrate utilization for *E. coli* and non-typhoidal *Salmonella* spp. For *Campylobacter* cultures, catalase, hippurate, oxidase, and nitrate reduction tests were performed. Bacterial isolates were tentatively identified by comparing positive biochemical test results with reference strains.

### Culture confirmation based on MALDI-TOF, PCR and 16S rRNA gene sequencing

All cultures were initially identified using MALDI-TOF (Bruker, Germany). Further, DNA extraction was performed using the QIAamp DNA Mini Kit (QIAGEN) following the manufacturer’s protocol for Gram-negative bacteria. Molecular confirmation was carried out using a multiplex PCR assay to identify both the *Campylobacter* genus and the two major species, *C. coli* and *C. jejuni* employing genus and species-specific primers (Wang et al., 2002). Primers used for *E. coli* and non-typhoidal *Salmonella* species are listed in **Table 1**. Appropriate positive and negative controls were included during the PCR reaction set up. Cultures confirmed by genus or species-specific primers were further identified by 16S rRNA gene sequencing (primer details in **Table 1**) using 3500XL Genetic Analyzer (Applied Biosystems, USA).

**Table 1:** Details of target genes and primers used for the amplification of *Campylobacter* spp., *Escherichia coli*, *Salmonella* spp., and other bacterial isolates from poultry farms and live bird markets.

Organism	Target	Forward primer (5’->3’ on plus strand)	Reverse primer (5’->3’ on minus strand)
*Campylobacter* spp.	23S rRNA	TATACCGGTAAGGAGTGCTGGAG	ATCAATTAACCTTCGAGCACCG
*C. coli*	*glyA gene*	GTAAAACCAAAGCTTATCGTG	TCCAGCAATGTGTGCAATG
*C. jejuni*	*hipO gene*	ACTTCTTTATTGCTTGCTGC	GCCACAACAAGTAAAGAAGC
*Escherichia coli*	adk gene	ATTCTGCTTGGCGCTCCGGG	CCGTCAACTTTCGCGTATTT
*Salmonella* spp.,	Non-typhoidal *Salmonella*	AGCCAACCATTGCTAAATTGGCGCA	GGTAGAAATTCCCAGCGGGTACTG
Universal primer for bacteria	16S rRNA gene	AGAGTTTGATCCTGGCTCAG	GGTTACCTTGTTACGACTT

### Antimicrobial susceptibility testing

Antibiotic susceptibility test (AST) was performed for *C. coli* (n=40), *C. jejuni* (n=21) and *E. coli* (n=77) using the Kirby-Bauer disc diffusion method (Bauer et al., 1966). AST was conducted on Mueller Hinton agar (supplemented with 5% sheep blood for *Campylobacter* spp.) (Weinstein and Lewis, 2020). A total of 34 antibiotics for *Campylobacter* spp. and 35 for *E. coli*, belonging to nine different antibiotic classes were used (HiMedia, India). A single colony was inoculated into culture specific growth medium and incubated for 18 to 24 hours for *E. coli* and 48 hours for *C. coli/C. jejuni* at culture specific incubation temperature. After incubation, when the OD reached 0.5 McFarland turbidity standard, 0.22 ml of culture was spread on Muller Hinton agar plates (200 mm diameter) using a sterile glass spreader. Antimicrobial disks (Himedia, India) were placed onto the surface of the inoculated agar plate using sterile forceps, and each disk was pressed down to ensure full contact. Plates were inverted and incubated within 15 minutes of placing the disks (at 42°C, in CO_2_ incubator for *C. coli/C. jejuni*). After 16 to 18 hours of incubation, the diameter of the zones of inhibition was measured to the nearest millimeter using a zone scale. *E. coli* ATCC 25922 was used as a reference for comparative analysis to ensure test repeatability and reliability. The CLSI (2020) guidelines were used to classify *C. jejuni*, *C. coli* and *E. coli* isolates as susceptible (S), intermediate (I), or resistant (R) (**Supplementary Table 2**). The multiple antibiotic resistance (MAR) index was calculated according to Krumperman (1983) as the ratio of total number of antibiotics to which the given isolate was resistant to the total number of antibiotics tested. Resistant isolates were categorized as multi-drug resistant (MDR: resistance to a minimum of one agent in more than three antimicrobial categories), extensively drug resistant (XDR: resistant to at least one antibiotic in all categories, except two) or pan-drug resistant (PDR: resistant to all tested antimicrobial agents).

### Statistical analysis

Statistical analysis was performed using GraphPad Prism (Version 10). *Campylobacter* prevalence across eight cities was compared using the Chi-square (χ²) test and Fisher’s extract test according to sample size. Statistical significance was determined for positive samples across city and bird type at p value = 0.05, and results with p < 0.05 were considered statistically significant.

## Results

As of March 2020, Gujarat comprised 3,211 poultry farms (as of March 2020) and following comprehensive surveillance, data were obtained from 31 out of 33 districts. Based on poultry density and distribution mapping eight cities, namely Ahmedabad, Bharuch, Bhuj, Godhra, Himatnagar, Rajkot, Surat, and Vadodara were selected for sample collection (**Figure 1**).

**Figure 1:**
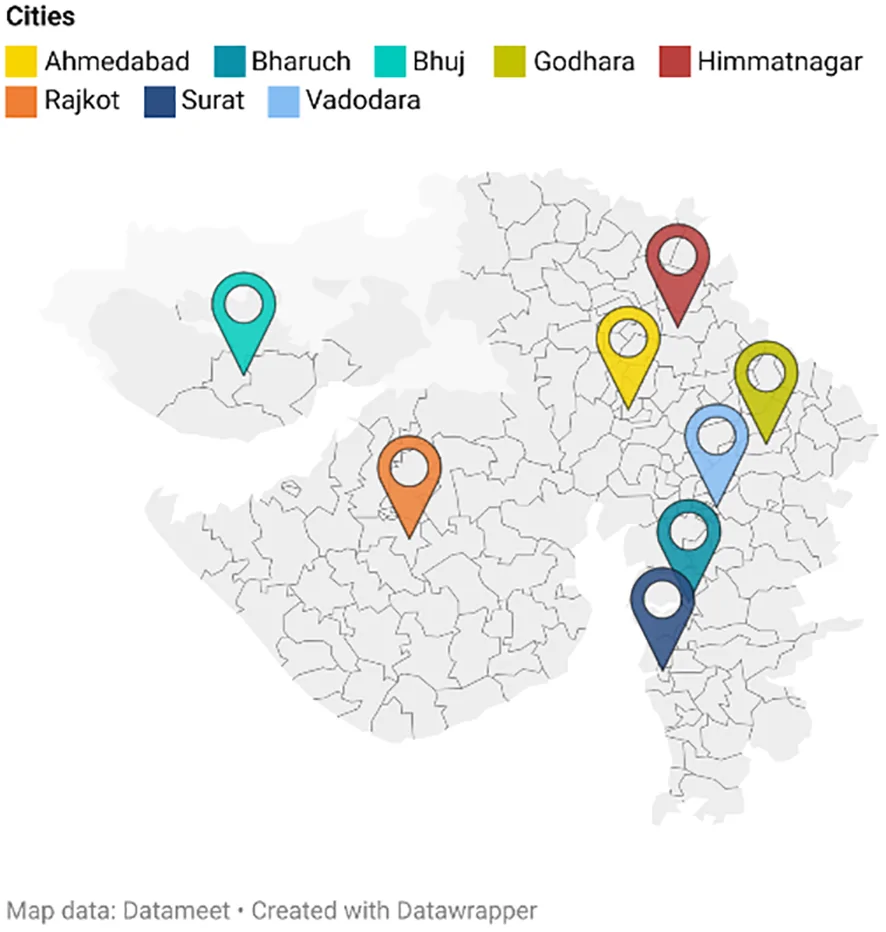
Sampling locations of live bird shops and poultry farms in eight cities across Gujarat.

### Isolation and biochemical characterization of microorganisms from collected samples

A total of 750 chicken cecal samples were collected from chicken shops and farms, subsequently processed for culture isolation. On mCCDA, 380 isolates produced grayish color colonies. Based on colony characteristics, 95 isolates were screened further for biochemical characterization including oxidase, catalase and hippurate tests. Isolates showing all three tests positive were tentatively sorted as belonging to *Campylobacter* genus. Using multiplex PCR optimized for *Campylobacter* genus specific 23S rRNA primers (650 bp) and species-specific primers for *C. jejuni* (hipO, 323 bp) and *C. coli* (glyA, 126 bp), 77 isolates were confirmed as *Campylobacter* spp. (**Table 1**; **Figure 2**). Concurrently, 150 environmental samples from poultry shops and farms were screened for the presence of non-typhoidal *Salmonella* (NTS). Based on colony morphology on MSRV and XLD agar plates, 52 isolates from poultry samples were selected for further confirmation. However, none of them were identified as NTS. Additionally, 150 chicken cloacal samples were processed for isolation and biochemical characterization of *Escherichia coli*. Using genus specific primers under optimized PCR conditions, a total of 95 samples, comprising 63 poultry shops and 32 farms were found positive for *E. coli* (**Table 2**; **Figure 3**).

**Table 2:** Sample details for the isolation of pathogens from poultry chicken, and poultry environment include the number of positive samples identified from each source.

Sample type	Targeted pathogens	Total sample collections	Positive
Chicken ceca	*C. coli* and *C. jejuni*	750	77
Chicken cloaca	*E. coli*	150	77
Poultry environmental swab	Non-typhoidal Salmonella	150	NIL

**Figure 2:**
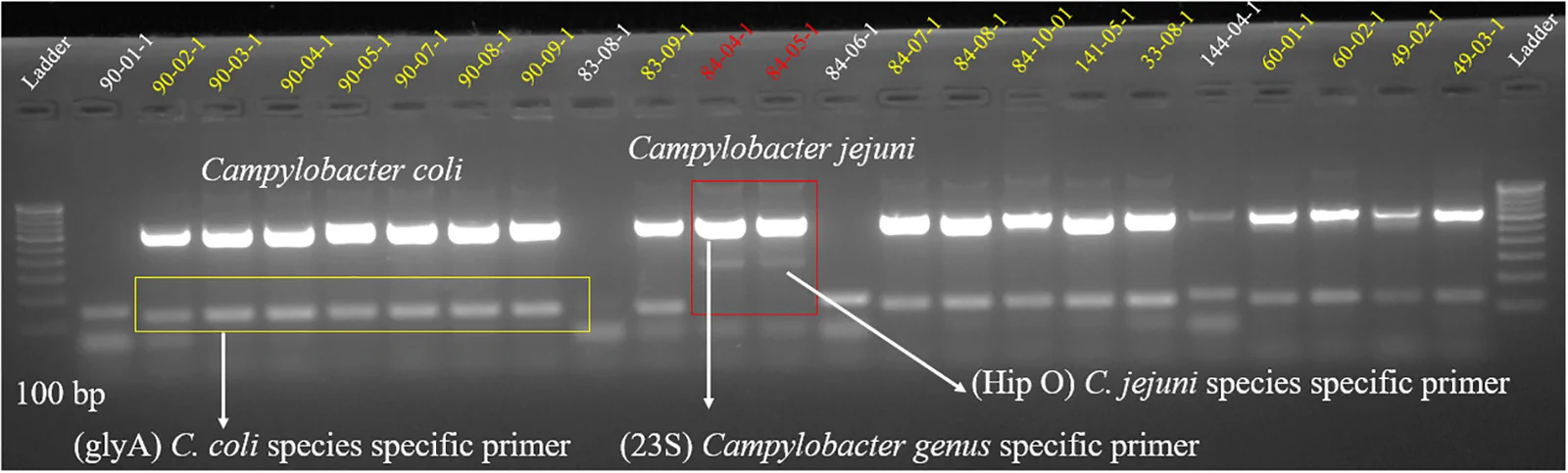
Multiplex PCR results showing the detection of *Campylobacter* spp. using the 23S rRNA primer, *C. jejuni* using the hipO primer, and *C. coli* using the glyA primer. The expected amplicon sizes were 650 bp for 23S rRNA gene, 323 bp for hipO and 126 bp for glyA.

**Figure 3:**
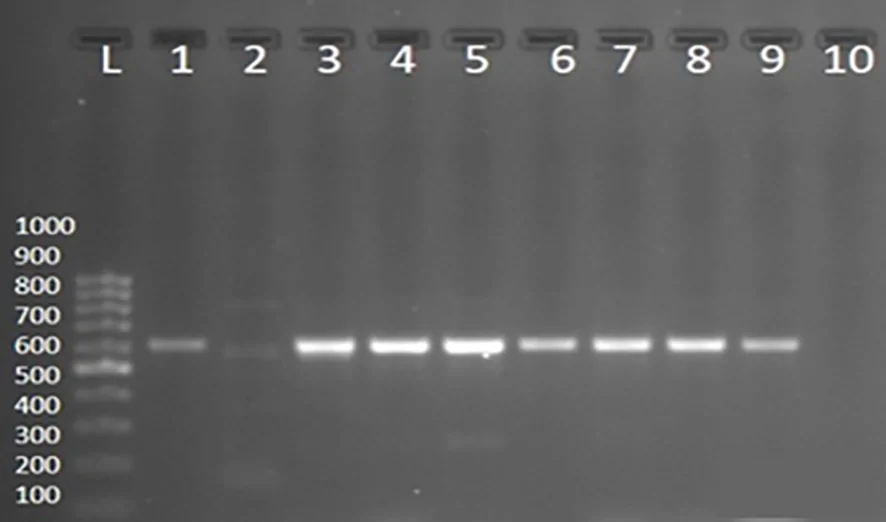
PCR gel image for amplification of specific primers for *Escherichia* genus. The expected product size is 584 bp. L indicates ladder (size 100 to 1000 bp) and sample 1 to 9 indicate positive *Escherichia coli* samples from chicken cloaca and 10 is no template control.

### Identification and prevalence of *Campylobacter* spp. and *E. coli*

For further confirmation of the isolates, microbial identification was performed by MALDI-TOF and 16S rRNA gene sequencing. Of the 77 *Campylobacter* isolates, 56 were identified as *C. coli* (7.46%) and 21 as *C. jejuni* (2.8%). For *E. coli*, 77 isolates were confirmed (51.33%, **Table 2**). The 16S rRNA sequences were submitted to NCBI and accession numbers are given in Supplementary File 4. The pattern of *C. coli* and *C. jejuni* prevalence varied across cities and poultry breeds (**Table 3**). The Chi-square test revealed a significant association between city and *C*. *coli* prevalence (χ²= 16.72, df= 7, p value= 0.0193), with the highest prevalence observed in Rajkot (13.04%) and the lowest in Himatnagar (1.11%) and Vadodara (1.25%). The highest prevalence of *C. coli* observed in bird type was broiler chicken from shop in Surat (17.5%) and in desi chickens from shops from Bhuj (16.7%). Fisher’s exact test demonstrated a highly significant association between bird type and *C. jejuni* positivity (p value= 0.0005), with 4.12% (21 out of 510 samples) of broiler samples testing positive compared to 0.00% in desi birds.

**Table 3:** City-wise distribution of *Campylobacter coli* and *Campylobacter jejuni* positivity across eight urban centers in Gujarat (n=750).

Source City	*C. coli*		*C. jejuni*		Total samples (n=750)
	Positive	Negative	Positive	Negative	
Ahmedabad	8	97	5	100	105
Bharuch	6	84	1	89	90
Bhuj	5	60	5	60	65
Godhra	9	91	2	98	100
Himatnagar	1	89	0	90	90
Rajkot	15	100	7	108	115
Surat	11	94	0	105	105
Vadodara	1	79	1	79	80

Overall, *C. coli* was more prevalent (7.5%) than *C. jejuni* (2.8%). Values represent absolute count of positive and negative samples per species for each city, with total sample size indicated.

### City wise *Campylobacter* species prevalence in broiler and desi breeds

In farm raised broilers (n=250), *C. jejuni* (12/250, 4.8%) was detected more frequently than *C. coli* (6/250, 2.4%). The majority of farm-level positives were observed in Rajkot, which accounted for 7 *C. jejuni* and 4 *C. coli* cases. Other cities with notable farm positivity included Ahmedabad (*C. jejuni*: 3/35) and Godhra (*C. jejuni*: 2/40; *C. coli*: 1/40). Farms in Bharuch, Bhuj, Himatnagar, Surat, and Vadodara showed minimal or zero *Campylobacter* positivity.

In broiler samples sourced from live bird market shops, the prevalence pattern reversed, with *C. coli* (26/260, 10.0%) being far more prevalent than *C. jejuni* (4/260, 1.5%). Surat recorded the highest *C. coli* positivity in this category (7/40), followed by Rajkot (8/30) and Godhra (5/35). *C. jejuni* in broiler shops was sporadic, detected only in Ahmedabad (2/35), Bharcuh (1/30), and Vadodara (1/30). No *C. jejuni* was detected in broiler shops in Bhuj, Godhra, Himatnagar, Rajkot, or Surat. There were some farm and shop sites (ID 130, 83, 84, 49) where from same/different birds, both types of *Campylobacter* species were observed.

Samples (n=240) from Desi (indigenous) birds sold in shops showed a notable absence of *C. jejuni* (0/240). However, *C. coli* prevalence was substantial with 10% (24/240). The highest *C. coli* positivity in Desi birds was observed in Ahmedabad (5/35), Bhuj (5/30), and Surat (4/30). However, Vadodara recorded zero *C. coli* positives in Desi birds, and Himatnagar recorded only one positive (1/30, **Table 4**).

**Table 4:** Prevalence of *Campylobacter* spp. in poultry farm (broilers) and live bird market shop (broilers and desi) samples across eight cities of Gujarat (*Campylobacter* spp. prevalence, expressed as number samples positive/the total number of samples processed).

Source City	Broiler (Farm, n=250)		Broiler (Shop, n=260)		Desi (Shop, n=240)	
	*C. jejuni*	*C. coli*	*C. jejuni*	*C. coli*	*C. jejuni*	*C. coli*
Ahmedabad	3/35	0/35	2/35	3/35	0/35	5/35
Bharuch	0/30	1/30	1/30	2/30	0/30	3/30
Bhuj	5/5	0/5	0/30	0/30	030	5/30
Godhra	2/40	1/40	0/35	5/35	0/25	3/25
Himatnagar	0/30	0/30	0/30	0/30	0/30	1/30
Rajkot	7/55	4/55	0/30	8/30	0/30	3/30
Surat	0/35	0/35	0/40	7/40	0/30	4/30
Vadodara	0/20	0/20	1/30	1/30	0/30	0/30

For *E. coli*, the highest prevalence rates (up to 8%) were observed in samples from Rajkot and Vadodara, whereas Bharuch and Bhuj reported less than 5% positivity. City, location and bird wise abundance and distribution of all isolates is presented in **Figure 4**.

**Figure 4:**
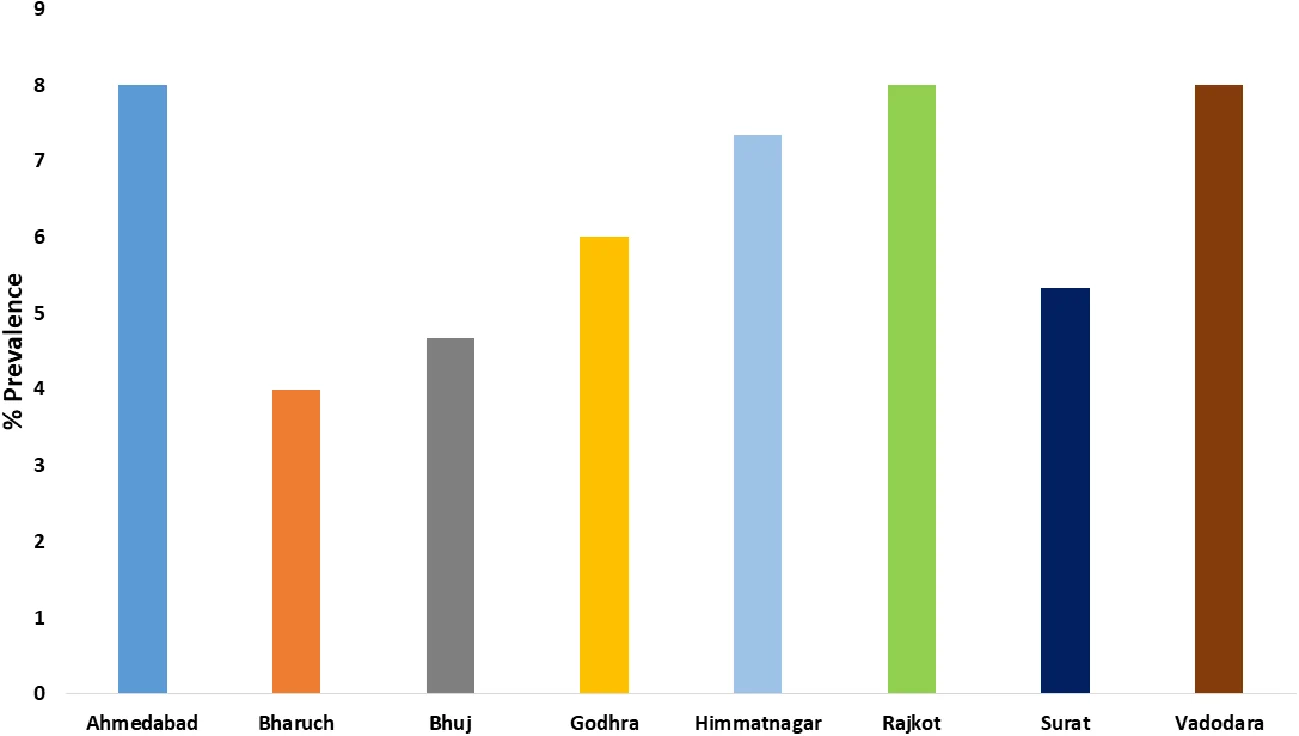
Percent prevalence of *E. coli* as confirmed by Sanger sequencing in poultry farms and live bird market across different cities of Gujarat.

### Antimicrobial susceptibility of isolates

Antimicrobial resistance was assessed for 61 *Campylobacter* spp. isolates and the results obtained for each antibiotic class are presented in **Figure 5**. The highest resistance levels (>80%) were observed across all isolates against piperacillin/tazobactam, ampicillin/sulbactam, novobiocin, cephalothin, co-trimoxazole, cefazolin, gatifloxacin, azithromycin, penicillin and tetracycline (**Figure 5**). All 77 *E. coli* isolates were found resistant to vancomycin belonging to class glycopeptide. In contrast, 40%, 38% and 35% of isolates were found susceptible to amikacin, streptomycin, and gentamicin (**Figure 6**). Among the 61 *Campylobacter* spp. isolates, 34 (55.74%) were classified as multidrug resistant (MDR), 24 (39.34%) as extensively drug resistant (XDR), and 3 (4.92%) as pan drug resistant (PDR). For 77 *E. coli* isolates, 23 (29.87%) were MDR, 47 (61.03%) were XDR, and 7 (9.09%) were PDR. Notably, 60 out of 61 *Campylobacter* and all *E. coli* isolates exhibited MAR indices greater than 0.2, indicating a significant risk of antimicrobial resistance genes dissemination in environments in which antibiotics are regularly administered.

**Figure 5:**
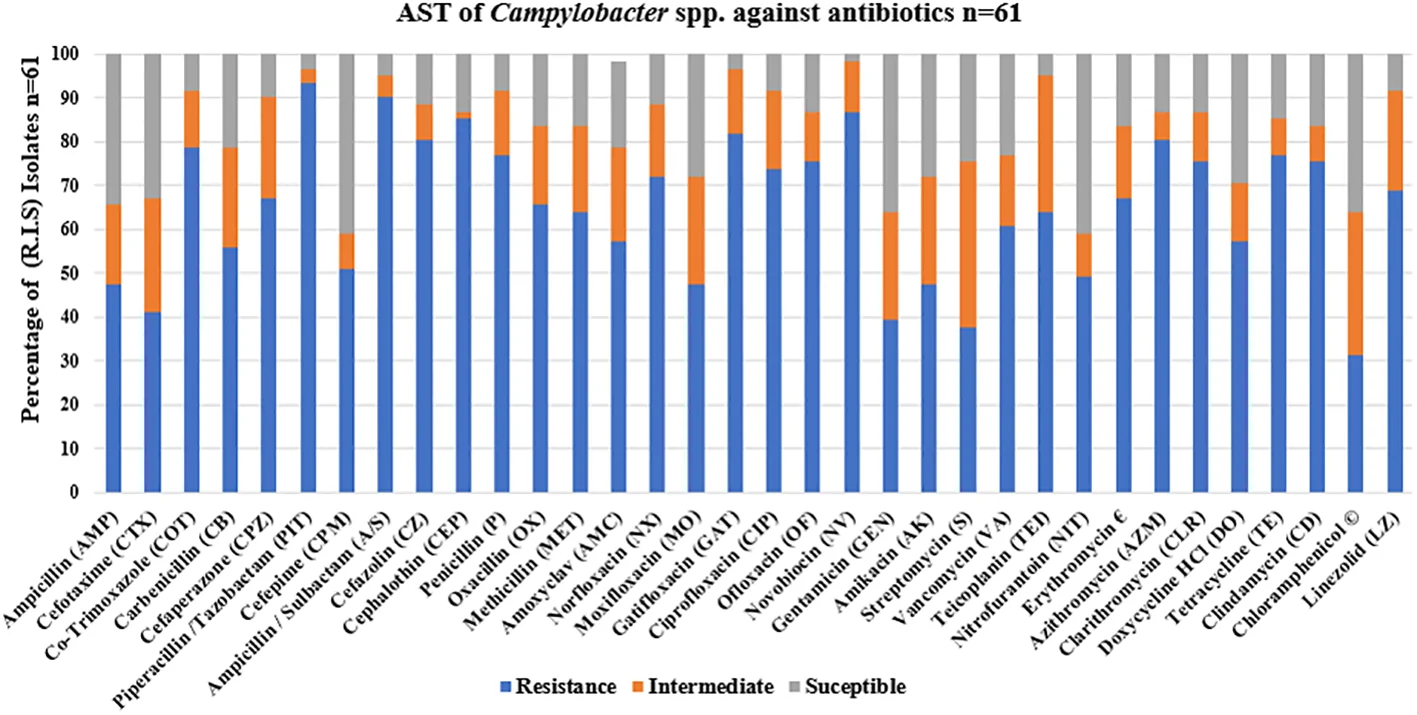
Overall prevalence percentage of antibiotics among 61 *Campylobacter* isolates (n=61) by disc diffusion assay. R, I and S indicate resistant, intermediate and susceptible as per the CLSI guidelines, respectively.

**Figure 6:**
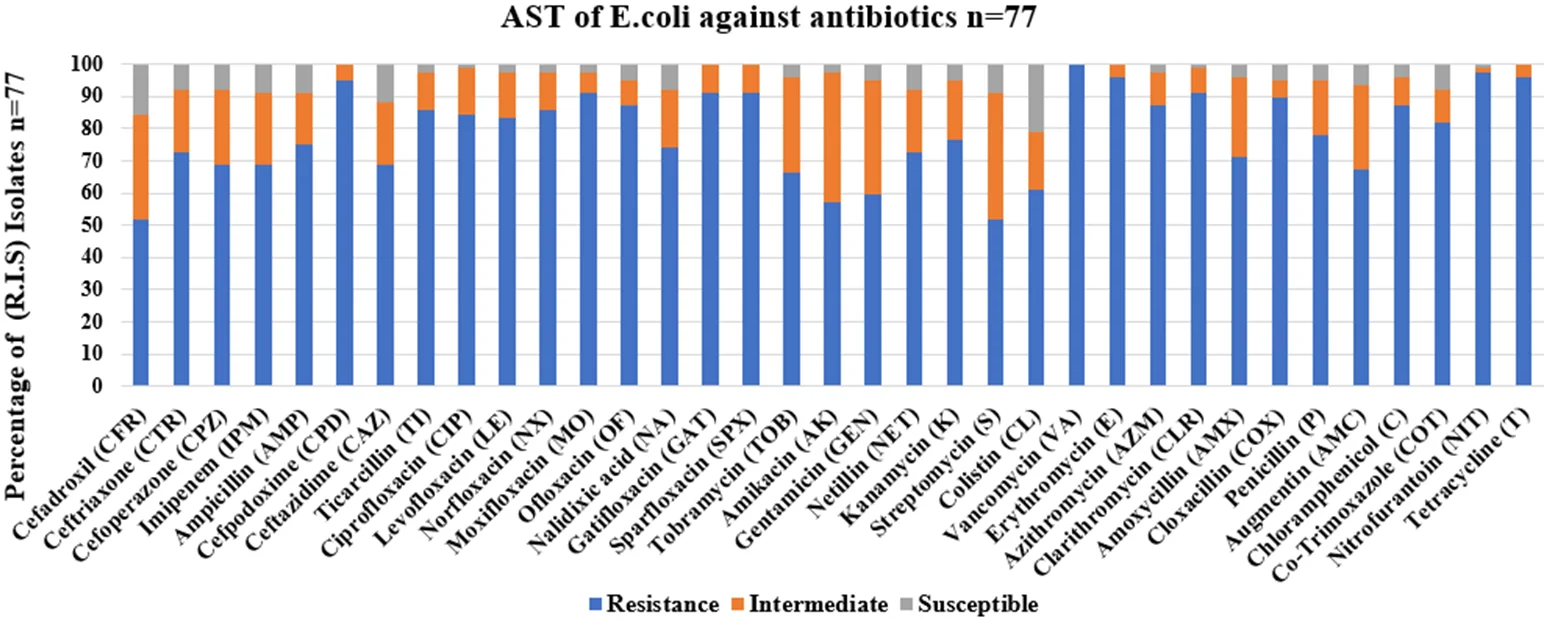
Overall prevalence percentage of antibiotics among 77 *E. coli* isolate by disc diffusion assay. R, I and S indicate resistant, intermediate and susceptible as per the CLSI guidelines, respectively.

## Discussion

This is the first study in Gujarat to concurrently evaluate *Campylobacter coli*, *Campylobacter jejuni*, and *Escherichia coli* across poultry food chain within the Gujarat region. By generating prevalence data and phenotypic AMR profiles against clinically relevant antibiotics, this research establishes a region-specific baseline to inform targeted One Health surveillance and intervention strategies. Infected birds carrying *Campylobacter* species harbor these microorganisms at primary colonization sites such as the caeca, cloaca, and colon where a high load of these pathogenic bacteria accumulates in the gastrointestinal tract. When comparing the prevalence of *Campylobacter* spp. across chicken varieties, the desi breed exhibited higher occurrence of *C. coli* in comparison to broilers, whereas the reverse was observed for *C. jejuni*, which was not detected in any desi breed bird. The overall prevalence of *C. coli* and *C. jejuni* was found to be 7.46% and 2.8%, respectively, among 750 samples processed. Similar findings have been reported for *C. jejuni* in 11.63% and *C. coli* in 1.16% of chicken meat and carcass samples from India and other countries (Gupta et al., 2025; Singh et al., 2009; Kaakoush et al., 2015). For indigenously reared desi chicken varieties in India, a previous study by Rangaraju et al. (2022) depicted 13.27% prevalence of *Campylobacter* species which is higher than our findings. The predominance of *C. coli* (7.46%) over *C. jejuni* (2.8%) aligns with reports from tropical regions likely due to greater thermotolerance, desiccation resistance, and competitive advantage of *C. coli* in under stress conditions such as high stocking densities and heat stress common in intensive poultry systems. The relatively low prevalence compared to temperate countries may be due to limited environmental survival and region-specific management practices that reduce initial colonization. The significant inter-city variation in *Campylobacter* prevalence (χ² = 26.26, p= 0.0005) highlights geographical heterogeneity within Gujarat’s poultry sector. This variation likely reflects differences in poultry processing, climate, production intensity, biosecurity protocols, and market hygiene (Gourmelon et al., 2022). This underscores the need for region-specific surveillance.

The cosistent absence of *C. jejuni* in desi birds (0/240) alongside substantial *C. coli* burden (10.0%) is a consistent finding across all cities. This suggests an inherent breed resistance to *C. jejuni* colonization, possibly due to genetic differences in gut microbiota or mucosal immunity. Alternatively, free-range scavenging exposes desi birds to environmental *C. coli* rather than the dense horizontal transmission of *C. jejuni* seen in commercial flocks (Chintoan-Uta et al., 2020). The prevalence of *Campylobacter* species was lower during the monsoon but increased from January to March in our study. This aligns with previous report indicating that broiler birds are frequently colonized by high numbers of *Campylobacter* species in the absence of clinical symptoms (Perez-Arnedo and Gonzalez-Fandos, 2019). Additionally, intensive commercial rearing systems may facilitate higher microbial colonization rates, possibly due to crowding, stress or feed-related factors, highlighting the need for improved management. The distribution patterns underscore the role of bird type, season, source, and city in *Campylobacter* spp. distribution. Antimicrobial resistance genes (ARGs) and drug resistance mechanisms in *C. coli* and *C. jejuni* isolates have been previously documented in studies worldwide covering antibiotic classes including beta-lactams, fluoroquinolones, aminoglycosides, non-beta-lactams, macrolides, tetracyclines, chloramphenicol, and oxazolidinones (Hoang et al., 2026; Dantroliya et al., 2025; Mbindyo et al., 2026; Popa et al., 2025a, 2025b; Wang et al., 2024). *Campylobacter* infections leading to endocarditis and pulmonary infections are typically treated with ampicillin/sulbactam in immunocompromised hosts (Schiaffino and Kosek, 2020). Therefore, the high resistance to these drugs underscored the urgent need for designing and implementing antibiotic usage policies with a One Health perspective to mitigate ARG dissemination across human-environment-animal chain. A detailed understanding of the genetic pathways for emerging AMR and increased virulence in pathogens are essential for possible intersections to effectively control and reduce the risk of zoonotic transmission to humans (Tripathi et al., 2025).

NTS *enterica* serovars prevalence in chicken rearing and processing habitats from farms and live bird markets in India have been documented only in a limited number of studies. The present study establishes the null prevalence of *Salmonella* spp. in environmental samples from poultry processing area. Previous studies conducted in India revealed that the occurrence of *Salmonella* in poultry slaughter samples was either similar or slightly higher (Kumar and Singh, 2014; Waghamare et al., 2018; Shivaning Karabasanavar et al., 2020). In contrast, considerably higher prevalence rates of *Salmonella* spp. have been reported in other parts of world from cloacal swabs of chicken, intestinal fluids, egg surface, visceral organ samples, and soil samples from chicken market (Paul et al., 2017; Hossain et al., 2021). A limitation of this study is that environmental surveillance was based on surface swab samples rather than bulk soil samples. Although surface swabbing is a well-established approach for monitoring poultry-associated environmental contamination, it may underestimate the presence of *Salmonella* if the organism is present in deeper soil layers or at low concentrations. Therefore, future studies incorporating both surface swabs and bulk soil samples may improve the sensitivity of *Salmonella* detection.

The prevalence rate of *E. coli* was 51.33% among 150 samples processed. *E. coli* prevalence did not differ significantly across eight cities (χ²= 6.214, df= 7, p= 0.515), suggesting a more uniform distribution of this organism within the study region. *E. coli* prevalence varied by location, with higher detection rates in Rajkot and Vadodara, while Bharuch and Bhuj had relatively lower rates. Antimicrobial susceptibility testing (AST) detected 100% resistance among all 77 isolates to the standard antibiotic vancomycin. This finding is clinically important as vancomycin is used in critical infection cases caused by Gram-positive bacteria as “last-line” antibiotics (Wijesekara et al., 2017). The rise of AMR in bacteria has become a noteworthy and widespread concern in the realm of public health. The implications of AMR encompass challenges such as the complexity or even the inability to effectively treat infections, leading to elevated rates of morbidity and mortality. While most *E. coli* strains are commensal gut microbiota in humans and animals, some toxin producing strains cause severe foodborne illness. The antimicrobial agents most frequently utilized by the farmers in the areas under investigation are enrofloxacine, neomycin, levofloxacin, oxytetracycline, gentamicin, amikacin, neofloxacine, and doxycycline belonging to the class fluoroquinolone, aminoglycoside and tetracycline. The higher antibiotic resistance among the studied pathogenic species may have emerged because of excessive overuse of antimicrobial agents such as prophylactic treatments to keep chicken healthy as feed additives (Abou-Jaoudeh et al., 2024). It has also been reported that resistance to several clinically relevant antibiotics can trigger or spread AMR to the other class of antibiotics even though they are not part of the feed as combined acquired resistance to several antibiotics (Udo et al., 2021). AMR is an alarming situation in the poultry industry due to improper administration of antimicrobial agents by farmers in the animal feed. Antimicrobial agents are easily accessible, and farmers directly purchase antibiotics without any recommendation from veterinarians in India. Excessive usage of antimicrobial agents may result in the alteration of the gut microflora or infection with drug resistant pathogens and can result in replacement of normal drug-sensitive bacteria as well as outbreak of untreatable infections (Brinkac et al., 2017; Singh et al., 2019). Continuous surveillance of AMR pattern in bacteria from poultry, the rearing environment and humans is crucial to guide the rational use of antimicrobials and effectively reduce AMR rates.

This study has a few limitations, such as potential selection bias due to voluntary participation, possible underestimation of pathogen prevalence from excluding clinically ill birds, a restricted sampling period that does not capture seasonal variations, and a limited environmental sampling scope that excluded water, feed and airborne sources for comprehensive transmission analysis.

## Conclusion

In light of increasing concern about antibiotic use and food safety, the present work reports the prevalence of *C. coli*, *C. jejuni*, and *E. coli* in poultry production system of Gujarat, India. Furthermore, we underscore the presence of antimicrobial resistance in these isolates. Therefore, the raw or improperly cooked meat can serve as a potential transmission vehicle for the spread of studied organisms and pose a risk of human infection. Moreover, the current study also highlights alarming levels of AMR in *Campylobacter* and *E. coli* species against several important clinical drugs which is a primary concern. This poses significant public health risks, as these resistant bacteria can be transmitted to humans via the food chain. These findings will help in formulation of the sustainable antibiotic usage policies and prescription practices for humans and poultry, guiding for improvement in food safety, productivity and economic outcomes. Coordinated actions by making policies are required to reduce or eliminate the risks posed by these pathogens as AMR can develop and spread at various stages within the food chain. To prevent diseases and their adverse health effects to humans and animals on a mass scale, it is imperative to identify threat, early warning signs and act quickly to maintain a healthier and safer environment. In conclusion, the findings emphasize the necessity of monitoring and regulating antibiotic use to ensure food safety and public health.

## Data Availability

The datasets presented in this study can be found in online repositories. The names of the repository/repositories and accession number(s) can be found in the article/**Supplementary Material**.
