# Supplementary material for: Prevalence and antimicrobial resistance profiles of Campylobacter coli, Campylobacter jejuni, and Escherichia coli in the poultry distribution network
Source: Front Antibiot. 2026 Sep 22;5:1875259. doi: 10.3389/frabi.2026.1875259 (PMC13640147; doi:10.3389/frabi.2026.1875259)
Supplement: Supplementary file 1 [file Table1.docx]

**Table 1**: Total 750 samples collected for the isolation of *Campylobacter* species from 8 cities of Gujarat. 1 to 100 samples were collected from different shops and 101 to 150 samples were collected from poultry farms. Total 77 samples were detected positive for the presence of *Campylobacter coli* or *Campylobacter* *jejuni*. Sample locations are mentioned in the table below.

| **Sr. No.** | **Sample ID** | **Scientific name** | **Location** | **Bird type** |
| --- | --- | --- | --- | --- |
| 1 | 46-06-1 | *Campylobacter coli* | Ahmedabad | Desi |
| 2 | 47-07-1 | *Campylobacter coli* | Ahmedabad | Desi |
| 3 | 90-02-1 | *Campylobacter coli* | Ahmedabad | Broiler |
| 4 | 90-03-1 | *Campylobacter coli* | Ahmedabad | Broiler |
| 5 | 90-04-1 | *Campylobacter coli* | Ahmedabad | Broiler |
| 6 | 90-07-1 | *Campylobacter coli* | Ahmedabad | Desi |
| 7 | 90-08-1 | *Campylobacter coli* | Ahmedabad | Desi |
| 8 | 150-01-1 | *Campylobacter jejuni* | Ahmedabad | Broiler |
| 9 | 37-03-1 | *Campylobacter jejuni* | Ahmedabad | Broiler |
| 10 | 47-09-1 | *Campylobacter coli* | Ahmedabad | Desi |
| 11 | 149-01-1 | *Campylobacter jejuni* | Ahmedabad | Broiler |
| 12 | 149-05-1 | *Campylobacter jejuni* | Ahmedabad | Broiler |
| 13 | 37-05-1 | *Campylobacter jejuni* | Ahmedabad | Broiler |
| 14 | 51-06-1 | *Campylobacter coli* | Bharuch | Desi |
| 15 | 51-07-1 | *Campylobacter coli* | Bharuch | Desi |
| 16 | 51-08-1 | *Campylobacter coli* | Bharuch | Desi |
| 17 | 134-03-1 | *Campylobacter coli* | Bharuch | Broiler |
| 18 | 49-02-1 | *Campylobacter coli* | Bharuch | Broiler |
| 19 | 49-03-1 | *Campylobacter coli* | Bharuch | Broiler |
| 20 | 49-04-1 | *Campylobacter jejuni* | Bharuch | Broiler |
| 21 | 81-07-1 | *Campylobacter coli* | Bhuj | Desi |
| 22 | 83-01-1 | *Campylobacter jejuni* | Bhuj | Broiler |
| 23 | 83-02-3 | *Campylobacter jejuni* | Bhuj | Broiler |
| 24 | 83-03-1 | *Campylobacter jejuni* | Bhuj | Broiler |
| 25 | 83-09-1 | *Campylobacter coli* | Bhuj | Desi |
| 26 | 84-04-1 | *Campylobacter jejuni* | Bhuj | Broiler |
| 27 | 84-05-1 | *Campylobacter jejuni* | Bhuj | Broiler |
| 28 | 84-07-1 | *Campylobacter coli* | Bhuj | Desi |
| 29 | 84-08-1 | *Campylobacter coli* | Bhuj | Desi |
| 30 | 84-10-1 | *Campylobacter coli* | Bhuj | Desi |
| 31 | 141-01-1 | *Campylobacter coli* | Godhra | Broiler |
| 32 | 22-03-1 | *Campylobacter coli* | Godhra | Broiler |
| 33 | 22-05-1 | *Campylobacter coli* | Godhra | Broiler |
| 34 | 22-07-1 | *Campylobacter coli* | Godhra | Desi |
| 35 | 22-08-1 | *Campylobacter coli* | Godhra | Desi |
| 36 | 22-09-1 | *Campylobacter coli* | Godhra | Desi |
| 37 | 23-02-1 | *Campylobacter coli* | Godhra | Broiler |
| 38 | 23-03-1 | *Campylobacter coli* | Godhra | Broiler |
| 39 | 23-05-1 | *Campylobacter coli* | Godhra | Broiler |
| 40 | 142-03-1 | *Campylobacter jejuni* | Godhra | Broiler |
| 41 | 142-04-1 | *Campylobacter jejuni* | Godhra | Broiler |
| 42 | 33-08-1 | *Campylobacter coli* | Himatnagar | Desi |
| 43 | 128-04-2 | *Campylobacter coli* | Rajkot | Broiler |
| 44 | 128-01-2 | *Campylobacter coli* | Rajkot | Broiler |
| 45 | 130-02-3 | *Campylobacter jejuni* | Rajkot | Broiler |
| 46 | 130-03-1 | *Campylobacter jejuni* | Rajkot | Broiler |
| 47 | 130-04-1 | *Campylobacter coli* | Rajkot | Broiler |
| 48 | 130-04-2 | *Campylobacter coli* | Rajkot | Broiler |
| 49 | 130-05-1 | *Campylobacter jejuni* | Rajkot | Broiler |
| 50 | 129-05-1 | *Campylobacter jejuni* | Rajkot | Broiler |
| 51 | 129-01-2 | *Campylobacter jejuni* | Rajkot | Broiler |
| 52 | 129-01-3 | *Campylobacter jejuni* | Rajkot | Broiler |
| 53 | 129-02-3 | *Campylobacter jejuni* | Rajkot | Broiler |
| 54 | 61-03-2 | *Campylobacter coli* | Rajkot | Broiler |
| 55 | 61-04-2 | *Campylobacter coli* | Rajkot | Broiler |
| 56 | 132-02-1 | *Campylobacter coli* | Rajkot | Broiler |
| 57 | 137-02-1 | *Campylobacter coli* | Rajkot | Broiler |
| 58 | 61-07-1 | *Campylobacter coli* | Rajkot | Desi |
| 59 | 61-08-1 | *Campylobacter coli* | Rajkot | Desi |
| 60 | 61-09-1 | *Campylobacter coli* | Rajkot | Desi |
| 61 | 132-05-1 | *Campylobacter coli* | Rajkot | Broiler |
| 62 | 130-02-1 | *Campylobacter coli* | Rajkot | Broiler |
| 63 | 60-01-1 | *Campylobacter coli* | Rajkot | Broiler |
| 64 | 60-02-1 | *Campylobacter coli* | Rajkot | Broiler |
| 65 | 68-07-1 | *Campylobacter coli* | Surat | Desi |
| 66 | 68-01-1 | *Campylobacter coli* | Surat | Broiler |
| 67 | 68-03-1 | *Campylobacter coli* | Surat | Broiler |
| 68 | 72-01-1 | *Campylobacter coli* | Surat | Broiler |
| 69 | 72-02-1 | *Campylobacter coli* | Surat | Broiler |
| 70 | 72-03-1 | *Campylobacter coli* | Surat | Broiler |
| 71 | 72-04-1 | *Campylobacter coli* | Surat | Broiler |
| 72 | 72-05-2 | *Campylobacter coli* | Surat | Broiler |
| 73 | 77-07-1 | *Campylobacter coli* | Surat | Desi |
| 74 | 77-08-1 | *Campylobacter coli* | Surat | Desi |
| 75 | 77-09-1 | *Campylobacter coli* | Surat | Desi |
| 76 | 08-02-1 | *Campylobacter coli* | Vadodara | Broiler |
| 77 | 08-05-1 | *Campylobacter jejuni* | Vadodara | Broiler |

**Table 2**: 150 samples screened for the isolation of *E. coli* and 77 samples from chicken cloaca were positive. Sample locations are mentioned in the table below.

| **Sr. No.** | **Sample ID** | **Scientific name** | **Location** | **Bird type** |
| --- | --- | --- | --- | --- |
| 1 | 111-01 | *Escherichia coli* | Ahmedabad | Broiler |
| 2 | 146-01 | *Escherichia coli* | Ahmedabad | Broiler |
| 3 | 147-01 | *Escherichia coli* | Ahmedabad | Broiler |
| 4 | 149-01 | *Escherichia coli* | Ahmedabad | Broiler |
| 5 | 150-01 | *Escherichia coli* | Ahmedabad | Broiler |
| 6 | 42-06 | *Escherichia coli* | Ahmedabad | Desi |
| 7 | 46-01 | *Escherichia coli* | Ahmedabad | Broiler |
| 8 | 46-06 | *Escherichia coli* | Ahmedabad | Desi |
| 9 | 90-01 | *Escherichia coli* | Ahmedabad | Broiler |
| 10 | 90-06 | *Escherichia coli* | Ahmedabad | Desi |
| 11 | 36-01 | *Escherichia coli* | Ahmedabad | Broiler |
| 12 | 36-06 | *Escherichia coli* | Ahmedabad | Desi |
| 13 | 145-01 | *Escherichia coli* | Bharuch | Broiler |
| 14 | 49-01 | *Escherichia coli* | Bharuch | Broiler |
| 15 | 56-06 | *Escherichia coli* | Bharuch | Desi |
| 16 | 58-01 | *Escherichia coli* | Bharuch | Broiler |
| 17 | 58-06 | *Escherichia coli* | Bharuch | Desi |
| 18 | 59-01 | *Escherichia coli* | Bharuch | Broiler |
| 19 | 79-06 | *Escherichia coli* | Bhuj | Desi |
| 20 | 80-01 | *Escherichia coli* | Bhuj | Broiler |
| 21 | 81-06 | *Escherichia coli* | Bhuj | Desi |
| 22 | 82-01 | *Escherichia coli* | Bhuj | Broiler |
| 23 | 83-01 | *Escherichia coli* | Bhuj | Broiler |
| 24 | 83-06 | *Escherichia coli* | Bhuj | Desi |
| 25 | 84-01 | *Escherichia coli* | Bhuj | Broiler |
| 26 | 139-01 | *Escherichia coli* | Godhra | Broiler |
| 27 | 140-01 | *Escherichia coli* | Godhra | Broiler |
| 28 | 142-01 | *Escherichia coli* | Godhra | Broiler |
| 29 | 104-01 | *Escherichia coli* | Godhra | Broiler |
| 30 | 15-01 | *Escherichia coli* | Godhra | Broiler |
| 31 | 22-01 | *Escherichia coli* | Godhra | Broiler |
| 32 | 17-01 | *Escherichia coli* | Godhra | Broiler |
| 33 | 19-01 | *Escherichia coli* | Godhra | Broiler |
| 34 | 19-06 | *Escherichia coli* | Godhra | Desi |
| 35 | 112-01 | *Escherichia coli* | Himatnagar | Broiler |
| 36 | 121-01 | *Escherichia coli* | Himatnagar | Broiler |
| 37 | 122-01 | *Escherichia coli* | Himatnagar | Broiler |
| 38 | 136-01 | *Escherichia coli* | Himatnagar | Broiler |
| 39 | 29-01 | *Escherichia coli* | Himatnagar | Broiler |
| 40 | 29-06 | *Escherichia coli* | Himatnagar | Desi |
| 41 | 30-01 | *Escherichia coli* | Himatnagar | Broiler |
| 42 | 31-01 | *Escherichia coli* | Himatnagar | Broiler |
| 43 | 31-06 | *Escherichia coli* | Himatnagar | Desi |
| 44 | 32-06 | *Escherichia coli* | Himatnagar | Desi |
| 45 | 33-06 | *Escherichia coli* | Himatnagar | Desi |
| 46 | 123-01 | *Escherichia coli* | Rajkot | Broiler |
| 47 | 124-01 | *Escherichia coli* | Rajkot | Broiler |
| 48 | 125-01 | *Escherichia coli* | Rajkot | Broiler |
| 49 | 126-01 | *Escherichia coli* | Rajkot | Broiler |
| 50 | 131-01 | *Escherichia coli* | Rajkot | Broiler |
| 51 | 132-01 | *Escherichia coli* | Rajkot | Broiler |
| 52 | 137-01 | *Escherichia coli* | Rajkot | Broiler |
| 53 | 60-01 | *Escherichia coli* | Rajkot | Broiler |
| 54 | 60-06 | *Escherichia coli* | Rajkot | Desi |
| 55 | 61-02 | *Escherichia coli* | Rajkot | Broiler |
| 56 | 66-01 | *Escherichia coli* | Rajkot | Broiler |
| 57 | 67-01 | *Escherichia coli* | Rajkot | Broiler |
| 58 | 114-01 | *Escherichia coli* | Surat | Broiler |
| 59 | 117-01 | *Escherichia coli* | Surat | Broiler |
| 60 | 144-01 | *Escherichia coli* | Surat | Broiler |
| 61 | 72-06 | *Escherichia coli* | Surat | Desi |
| 62 | 73-06 | *Escherichia coli* | Surat | Desi |
| 63 | 75-01 | *Escherichia coli* | Surat | Broiler |
| 64 | 77-01 | *Escherichia coli* | Surat | Broiler |
| 65 | 71-06 | *Escherichia coli* | Surat | Desi |
| 66 | 76-01 | *Escherichia coli* | Surat | Broiler |
| 67 | 102-01 | *Escherichia coli* | Vadodara | Broiler |
| 68 | 106-01 | *Escherichia coli* | Vadodara | Broiler |
| 69 | 002-01 | *Escherichia coli* | Vadodara | Broiler |
| 70 | 002-06 | *Escherichia coli* | Vadodara | Desi |
| 71 | 003-01 | *Escherichia coli* | Vadodara | Broiler |
| 72 | 005-06 | *Escherichia coli* | Vadodara | Desi |
| 73 | 008-06 | *Escherichia coli* | Vadodara | Desi |
| 74 | 10-01 | *Escherichia coli* | Vadodara | Broiler |
| 75 | 13-01 | *Escherichia coli* | Vadodara | Broiler |
| 76 | 001-01 | *Escherichia coli* | Vadodara | Broiler |
| 77 | 14-01 | *Escherichia coli* | Vadodara | Broiler |
